# Supplementary material for: On the hypercomplex numbers and normed division algebras in all dimensions: A unified multiplication
Source: PLoS One. 2024 Oct 25;19(10):e0312502. doi: 10.1371/journal.pone.0312502 (PMC11508080; doi:10.1371/journal.pone.0312502)
Supplement: Attachment — Submitted filename: hypercomplex2-plosone.pdf [file pone.0312502.s001.pdf]

Comments to authors

The manuscript describes an extension of complex algebra to higher dimensions. Three dimensions are treated in detail. The proposal is very interesting and worth pursuing.

Minor corrections:

p.5 The spherical coordinate system is formally defined in terms of the trigonometric functions, in particular, $\phi = \arctan\left(\frac{b}{a}\right)$. It is only in computer science where 'tan2' is used. It would be better to use the formal definition and, if required, introduce the computational definition thereafter.

p.5 Eq. (7) does not follow from Eq. (1)-(2). The wording before Eq. (7) has to be careful to avoid misunderstandings.

p.7 Addition and multiplication are the two fundamental operations. Subtraction and division come from inverse elements. Thus, it is customary to define only two operations.

p.8 Figure 2 can be greatly improved. The trajectory is not clear in (b) and (c), where it begins/ends and which path it follows. In fact, maybe (c) could be deferred mentioning that the ZY plane and ZX planes exhibit similar behaviour for the appropriate values of ϕ.

p.9 Eq. (25) *This is the most important revision that is needed* (Later results will have to be modified accordingly). Let me explain:

Equations (4) to (6) have been carefully devised so that the θ domain is $(-\infty, \infty)$. Then Eq. (7) is the crucial coordinate definition. The $(-\infty, \infty)$ θ domain is needed so that the product is a closed operation. However, in (25) what happens if θ is larger than $\left|\frac{\pi}{2}\right|$?

$e^{i\phi}$ and $e^{j\theta}$ are different functions, the "Euler identity" type expression for the latter involve your $\cos\pi(\theta)$ and $\sin\pi(\theta)$ functions.

p.9 Reconsider your assertion that "This multiplication is continuous, meaning there are no discontinuities or jumps in the resulting sphere." Evaluate, for example the product $\left(e^{i\phi_1}e^{j\theta_1}\right) \times \left(e^{j\theta_2}\right)$, where $\theta_1 = 0.9\frac{\pi}{2}$ and $\theta_2 = 0.2\frac{\pi}{2}$. Also, reconsider Remark 2.

p.9 In line Eq. after Eq. (26) implies $\phi = 0$ mod π.

p.12 Remark 4 seems unnecessary and it has major problems. Notice that neutral element in the two operations is different.

p.12 Eq. (34), has to be revised. Remember that the series representation and the Euler identity are two different things. Since the domain of $\cos\pi(\theta)$ and $\sin\pi(\theta)$ is $(-\infty, \infty)$, problems arise since $e^{j\theta}$ domain has been restricted to $\pm\frac{\pi}{2}$ interval. But if the product is closed it has to be defined in $(-\infty, \infty)$, just as $\cos\pi(\theta)$ and $\sin\pi(\theta)$ are. It seems to me that you have to define the $e^{j\theta}$ series using (4) to (6). Strictly speaking you should then label this 'exponential' function differently, since it differs from the usual complex exponential.

p.14 Some of your assertions and/or proofs have to be carefully worked out. Take for example Proposition1, 8), let $g_1 = e^{i\frac{\pi}{4}}e^{j\frac{\pi}{4}} \neq 0$ and $g_2 = e^{i\frac{\pi}{4}}e^{j\frac{\pi}{4}} \neq 0$, what is the value of $g_1 g_2 = e^{i\frac{\pi}{2}}e^{j\frac{\pi}{2}}$?
