# Supplementary material for: On the hypercomplex numbers and normed division algebras in all dimensions: A unified multiplication
Source: PLoS One. 2024 Oct 25;19(10):e0312502. doi: 10.1371/journal.pone.0312502 (PMC11508080; doi:10.1371/journal.pone.0312502)
Supplement: Attachment — Submitted filename: PONE_RL_R1.pdf [file pone.0312502.s002.pdf]

September 16, 2024

Response Letter

PLOS ONE

Dear Editor:

First of all, please accept our thanks for devoting time and coordinating the reviews of the submitted manuscript Ref. No. PONE-D-24-24879 titled "On the hypercomplex numbers and normed division algebras in all dimensions: A unified multiplication" for possible publication in PLOS ONE. We submitted the manuscript on June 19, 2024 and received the review comments from the reviewers on August 31, 2024. We appreciate the time and effort you and the anonymous reviewers devoted to reviewing and providing useful comments to help us improve the quality of the manuscript.

We have done our best to address the comments of the reviewers. We, hereby, submit: (1) a response letter with detailed replies to each of the reviewers' comments and (2) a revised manuscript that we believe to have significantly improved after incorporating suggestions of the reviewers.

Please do thank the reviewers on our behalf for their time and effort. We also thank you for your work as the editor. We have also acknowledged editors and anonymous reviewers in the acknowledgments section of the revised manuscript.

Thanks and regards,
Authors

Response to Reviewers: PONE-D-24-24879

Title: *On the hypercomplex numbers and normed division algebras in all dimensions: A unified multiplication*

- **Reviewer #1:**

  Comments to authors The manuscript describes an extension of complex algebra to higher dimensions. Three dimensions are treated in detail. The proposal is very interesting and worth pursuing.

  Response: Authors would like to thank the reviewer for providing detailed comments in the previous review round that helped us improve the quality of the manuscript.

  Minor corrections:

  p.5 The spherical coordinate system is formally defined in terms of the trigonometric functions, in particular, $\phi = \arctan(\frac{b}{a})$. It is only in computer science where 'tan2' is used. It would be better to use the formal definition and, if required, introduce the computational definition thereafter.

  Response: As per suggestion of the reviewer, we have used $\phi = \arctan(\frac{b}{a})$, and introduced the computational definition thereafter.

  p.5 Eq. (7) does not follow from Eq. (1)-(2). The wording before Eq. (7) has to be careful to avoid misunderstandings.

  Response: As per suggestion of the reviewer, we have changed text before (7) to avoid any misunderstandings.

  p.7 Addition and multiplication are the two fundamental operations. Subtraction and division come from inverse elements. Thus, it is customary to define only two operations.

  Response: As per suggestion of the reviewer, we have defined addition and multiplication as two fundamental operations, and mentioned that subtraction and division come from inverse elements.

  p.8 Figure 2 can be greatly improved. The trajectory is not clear in (b) and (c), where it begins/ends and which path it follows. In fact, maybe (c) could be deferred mentioning that the ZY plane and ZX planes exhibit similar behaviour for the appropriate values of ϕ.

  Response: Based on the suggestion of the reviewer, we have revised Figure 2, specifically parts (b) and (c). In these updated figures, the left semicircle is depicted in blue, and the right semicircle is in red

to illustrate the trajectory. This modification highlights the path traced by $\exp(j\theta)$, which follows a semicircular trajectory, as indicated by the blue and red segments for the appropriate values of ϕ.

p.9 Eq. (25) *This is the most important revision that is needed* (Later results will have to be modified accordingly). Let me explain: Equations (4) to (6) have been carefully devised so that the θ domain is $(-\infty, \infty)$. Then Eq. (7) is the crucial coordinate definition. The $(-\infty, \infty)$ θ domain is needed so that the product is a closed operation. However, in (25) what happens if θ is larger than $|\frac{\pi}{2}|$?

$\exp(i\phi)$ and $\exp(j\theta)$ are different functions, the "Euler identity" type expression for the latter involve your $\cos\pi(\theta)$ and $\sin\pi(\theta)$ functions.

Response: Following the suggestion of reviewer, we have revised the text accordingly. In Equations (7) and (25), in general, $\phi \in \mathbb{R}$, $\theta \in \mathbb{R}$, $e^{i\phi}$ is 2π-periodic and $e^{j\theta}$ is π-periodic. To normalize these angles, the 2π modulo operation is applied to ϕ to restrict it to the principal range $[0, 2\pi)$. Similarly, the π modulo operation is used to adjust θ to lie within the principal range $[-\pi/2, \pi/2]$. Specifically, if $\theta \notin [-\pi/2, \pi/2]$, it is normalized to this range by adding or subtracting a multiple of π, i.e., by applying the transformation $\theta \mapsto \theta + n\pi$, where $n \in \mathbb{Z}$. Therefore, the azimuth angle ϕ is considered 2π-periodic, and the elevation angle θ is considered π-periodic as shown in Fig. 4.

p.9 Reconsider your assertion that "This multiplication is continuous, meaning there are no discontinuities or jumps in the resulting sphere." Evaluate, for example the product $(e^{i\phi_1}e^{j\theta_1}) \times (e^{j\theta_2})$, where $\theta_1 = 0.9\frac{\pi}{2}$ and $\theta_2 = 0.2\frac{\pi}{2}$. Also, reconsider Remark 2.

Response: Based on the suggestion of the reviewer, we have removed the statement. We have also revised Remark 2 accordingly.

p.9 In line Eq. after Eq. (26) implies $\phi = 0 \mod \pi$.

Response: Thank you for suggestion, we have noted it.

p.12 Remark 4 seems unnecessary and it has major problems. Notice that neutral element in the two operations is different.

Response: Thank you for suggestion, we have removed it.

p.12 Eq. (34), has to be revised. Remember that the series representation and the Euler identity are two different things. Since the domain of $\cos\pi(\theta)$ and $\sin\pi(\theta)$ is $(-\infty, \infty)$, problems arise since $e^{j\theta}$ domain has been restricted to $\pm\frac{\pi}{2}$ interval. But if the product is closed it has to be defined in $(-\infty, \infty)$, just a $\cos\pi(\theta)$ and $\sin\pi(\theta)$ are. It seems to me that you have to define the $e^{j\theta}$ series using (4) to (6). Strictly speaking you should then label this 'exponential' function differently, since it differs from the usual complex exponential.

Response: Thank you for suggestion, now we have revised domain of θ as $\theta \in \mathbb{R}$ in (34). Moreover, in general, $\phi \in \mathbb{R}$, $\theta \in \mathbb{R}$, $e^{i\phi}$ is 2π periodic and $e^{j\theta}$ is π periodic. Thus to normalize these angles, the 2π modulo operation is applied to ϕ to confine it within the principal range $[0, 2\pi)$, and the π modulo operation is used to adjust θ to lie within the principal range $[-\pi/2, \pi/2]$. This suggests that we can also consider $\theta \in [-\pi/2, \pi/2]$ in (34).

p.14 Some of your assertions and/or proofs have to be carefully worked out. Take for example Proposition1, 8), let $g_1 = e^{i\frac{\pi}{4}}e^{j\frac{\pi}{4}} \neq 0$ and $g_2 = e^{i\frac{\pi}{4}}e^{j\frac{\pi}{4}} \neq 0$, what is the value of $g_1g_2 = e^{i\frac{\pi}{2}}e^{j\frac{\pi}{2}}$?

Response: There is a persistent nonuniqueness in representations that arises in specific scenario within the spherical coordinate system (SCS): Z-axis ($\theta = \pm\pi/2$): In this specific scenario where θ equals $\pi/2$ or $-\pi/2$, for a fixed r, the azimuthal angle ϕ can assume any value, as explicitly revealed by equation (2). Consequently, a vector situated along the Z-axis possesses an infinite array of representations as $(r, \phi, \pm\pi/2)$. This implies that vectors characterized by diverse values of r, ϕ, and θ can be aligned along the Z-axis by adjusting θ to $\pm\pi/2$. Once aligned along the Z-axis, alterations in the polar angle ϕ do not induce any alteration in the orientation of the vector. Thus, in these instances, manipulation of the values of one or more of the other coordinates can be undertaken without inducing displacement of the point in question.
Therefore, if $g_1 = e^{i\frac{\pi}{4}}e^{j\frac{\pi}{4}} \neq 0$ and $g_2 = e^{i\frac{\pi}{4}}e^{j\frac{\pi}{4}} \neq 0$, then $g_1g_2 = e^{i\frac{\pi}{2}}e^{j\frac{\pi}{2}} = j$. This is also same as $e^{i\phi}e^{j\frac{\pi}{2}} = j$ for any $\phi \in [0, 2\pi)$, which is explained in Eqns. (27), (28) and (29). Moreover, the azimuth angle ϕ in the expression $e^{i\phi}e^{j\frac{\pi}{2}} = j$ indicates that any subsequent variation in the elevation angle θ results in the displacement of the point from the Z-axis towards the direction specified by ϕ.

- **Reviewer #2:**

  Reviewer #2: In this paper the authors present a comprehensive generalization of complex numbers and the Euler identity in higher dimensions, shedding light on the geometric properties of vectors within these extended spaces. Moreover, they elucidated the practical applications of the proposed methodology as a viable alternative for expressing a quantum state through the multiplication of specified quantum states, thereby offering a potential complement to the established superposition paradigm. Importantly, they explored its utility in point cloud image processing. The paper is well supported by the examples and figures which puts life to the paper. The paper is well written and fits to the scope of the journal. I recommend acceptance.

  Response: Authors would like to thank the reviewer for recommendation to accept the paper, and devoting time to provide detailed comments and observations.

Thanks and Regards,
Pushpendra Singh
(On behalf of all authors)
