# Supplementary material for: On the hypercomplex numbers and normed division algebras in all dimensions: A unified multiplication
Source: PLoS One. 2024 Oct 25;19(10):e0312502. doi: 10.1371/journal.pone.0312502 (PMC11508080; doi:10.1371/journal.pone.0312502)
Supplement: Attachment — Submitted filename: hypercomplex2-plosone-rev2.pdf [file pone.0312502.s003.pdf]

Consider the complex set $\mathbb{C}$. The complex exponential series representation is

$$e^{i\phi} = \sum_{n=0}^{\infty} \frac{(i\phi)^n}{n!}, \tag{1}$$

where $i^2 = -1$. This series can be separated in two parts, one not involving i and one doing so

$$e^{i\phi} = \sum_{n=0}^{\infty} \frac{(i\phi)^{2n}}{(2n)!} + \sum_{n=0}^{\infty} \frac{(i\phi)^{2n+1}}{(2n+1)!} = \sum_{n=0}^{\infty} (-1)^n \frac{(\phi)^{2n}}{(2n)!} + i \sum_{n=0}^{\infty} (-1)^n \frac{(\phi)^{2n+1}}{(2n+1)!}.$$

Euler identity is

$$e^{i\phi} = \cos\phi + i\sin\phi.$$

Therefore, the series representation of the trigonometric functions is obtained

$$\cos\phi = \sum_{n=0}^{\infty} (-1)^n \frac{(\phi)^{2n}}{(2n)!}, \qquad i\sin\phi = i\sum_{n=0}^{\infty} (-1)^n \frac{(\phi)^{2n+1}}{(2n+1)!}. \tag{2}$$

Here, I am going to do some of the authors homework, but it should be clear that it is not my role. They should work out carefully their results.

- What I think is not correct is this (and consequences derived thereof):

Several times in the manuscript, "easily" is stated, and their result is incorrect. So perhaps it is not so "easily", for example that the "Euler identity" in (34) is derived.

The authors of this paper propose a modified set of trigonometric functions, $\cos\pi(\theta)$ and $\sin\pi(\theta)$ defined by eqs. (4) to (6).

However, the series expansion for $\cos\pi(\theta)$ in (34) is identical to the series expansion of $\cos\theta$ (replacing $\phi \to \theta$ is merely that, a variable replacement). Thus a contradiction arises, if their series are identical the functions are identical $\cos\pi(\theta) = \cos\theta$. But their eq. (4) states that $\cos\pi(\theta) = |\cos\theta|$, but $\cos\theta$ is not a positive definite function, hence the contradiction.

Another way to see this problem, authors claim $\cos\pi(\theta) = 1 - \frac{\theta^2}{2!} + \frac{\theta^4}{4!} - \frac{\theta^6}{6!} \ldots$ for all $\theta \in \mathbb{R}$ (different notation for what we stated here in (2)). Evaluate this series expression for $\theta = \pi$. The series converges to -1, but $\cos\pi(\theta)$ is semipositive definite! Therefore, the series is incorrect.

- If I am grasping the foundational ideas of the paper correctly, what is required is the following:

It seems to me that what the authors need to do is to define the series as: For all $\theta \in \mathbb{R}$,

$$\cos\pi(\theta) \equiv \begin{cases} \sum_{n=0}^{\infty} (-1)^n \frac{(\theta)^{2n}}{(2n)!} & \left[-\frac{\pi}{2}, \frac{\pi}{2}\right] \bmod \pi \\ -\sum_{n=0}^{\infty} (-1)^n \frac{(\theta)^{2n}}{(2n)!} & \left[\frac{\pi}{2}, \frac{3\pi}{2}\right] \bmod \pi \end{cases}$$

This series compared with the cosine series above (2), imply that $\cos\pi(\theta) = |\cos\theta|$, also that $\cos\pi(\theta)$ is semipositive definite, continuity of the function, derivative not defined at $\frac{\pi}{2}$ mod π, etc.

If $j^2 = -1$ and the product is associative, it follows that $j^3 = -j$ and $j^4 = 1$. In terms of the complex exponential function, defined here by (1),

$$\cos\pi(\theta) = \begin{cases} \mathrm{Re}\left(e^{i\theta}\right), & \left[-\frac{\pi}{2}, \frac{\pi}{2}\right] \text{ mod } \pi \\ \mathrm{Re}\left(-e^{i\theta}\right), & \left[\frac{\pi}{2}, \frac{3\pi}{2}\right] \text{ mod } \pi \end{cases}$$

Or, if the authors wish to define an exponential function, let me label it e_π, with series given by

$$\mathrm{Re}\left(e_\pi^{j\theta}\right) = \begin{cases} \mathrm{Re}\left(e^{i\theta}\right), & \left[-\frac{\pi}{2}, \frac{\pi}{2}\right] \text{ mod } \pi \\ \mathrm{Re}\left(-e^{i\theta}\right), & \left[\frac{\pi}{2}, \frac{3\pi}{2}\right] \text{ mod } \pi \end{cases}$$

then they could write

$$\cos\pi(\theta) = \mathrm{Re}\left(e_\pi^{j\theta}\right)$$

Notice that defining the π periodic exponential function e_π with the imaginary unit i or the imaginary unit j makes no difference.

I am deliberately leaving the other half of the definition undone. The authors have to figure out how to define the series representation so that they actually reproduce $\sin\pi(\theta)$ as defined in their eq. (5)-(6) [BTW, maybe there is a nicer way to define (5)-(6)]. They will then be able to define a π periodic complex exponential function ...
