# Supplementary material for: On the hypercomplex numbers and normed division algebras in all dimensions: A unified multiplication
Source: PLoS One. 2024 Oct 25;19(10):e0312502. doi: 10.1371/journal.pone.0312502 (PMC11508080; doi:10.1371/journal.pone.0312502)
Supplement: Attachment — Submitted filename: PONE_R2_RL.pdf [file pone.0312502.s004.pdf]

September 28, 2024

Response Letter

PLOS ONE

Dear Editor:

First, please accept our thanks for devoting time and coordinating the reviews of the submitted manuscript Ref. No. PONE-D-24-24879 titled "On the hypercomplex numbers and normed division algebras in all dimensions: A unified multiplication" for possible publication in PLOS ONE. We submitted the manuscript on June 19, 2024, and received review comments from the reviewers on August 31, 2024. The second round of review comments was received on September 23, 2024. We sincerely appreciate the time and effort by the anonymous reviewers dedicated to evaluating our manuscript and offering valuable feedback, which has significantly contributed to enhancing its quality.

We have done our best to address the reviewers' comments. We, hereby, submit: (1) a response letter with detailed replies to each of the reviewers' comments and (2) a revised manuscript that we believe to have significantly improved after incorporating the reviewers' suggestions.

Please do thank the reviewers on our behalf for their time and effort. We also thank you for your work as the editor. We have also acknowledged editors and anonymous reviewers in the acknowledgments section of the revised manuscript.

Thanks and regards,
Authors

Response to Reviewers: PONE-D-24-24879

Title: *On the hypercomplex numbers and normed division algebras in all dimensions: A unified multiplication*

Reviewer #1:

Consider the complex set $\mathbb{C}$. The complex exponential series representation is

$$e^{i\phi} = \sum_{n=0}^{\infty} \frac{(i\phi)^n}{n!}, \tag{1}$$

where $i^2 = -1$. This series can be separated in two parts, one not involving i and one doing so

$$e^{i\phi} = \sum_{n=0}^{\infty} \frac{(i\phi)^{2n}}{(2n)!} + \sum_{n=0}^{\infty} \frac{(i\phi)^{2n+1}}{(2n+1)!} = \sum_{n=0}^{\infty} (-1)^n \frac{(\phi)^{2n}}{(2n)!} + i \sum_{n=0}^{\infty} (-1)^n \frac{(\phi)^{2n+1}}{(2n+1)!}.$$

Euler identity is

$$e^{i\phi} = \cos\phi + i \sin\phi.$$

Therefore, the series representation of the trigonometric functions is obtained

$$\cos\phi = \sum_{n=0}^{\infty} (-1)^n \frac{(\phi)^{2n}}{(2n)!}, \qquad i \sin\phi = i \sum_{n=0}^{\infty} (-1)^n \frac{(\phi)^{2n+1}}{(2n+1)!}. \tag{2}$$

Here, I am going to do some of the authors homework, but it should be clear that it is not my role. They should work out carefully their results.

- What I think is not correct is this (and consequences derived thereof):

Several times in the manuscript, "easily" is stated, and their result is incorrect. So perhaps it is not so "easily", for example that the "Euler identity" in (34) is derived.

The authors of this paper propose a modified set of trigonometric functions, $\cos\pi(\theta)$ and $\sin\pi(\theta)$ defined by eqs. (4) to (6).

However, the series expansion for $\cos\pi(\theta)$ in (34) is identical to the series expansion of $\cos\theta$ (replacing $\phi \to \theta$ is merely that, a variable replacement). Thus a contradiction arises, if their series are identical the functions are identical $\cos\pi(\theta) = \cos\theta$. But their eq. (4) states that $\cos\pi(\theta) = |\cos\theta|$, but $\cos\theta$ is not a positive definite function, hence the contradiction.

Another way to see this problem, authors claim $\cos\pi(\theta) = 1 - \frac{\theta^2}{2!} + \frac{\theta^4}{4!} - \frac{\theta^6}{6!} \dots$ for all $\theta \in \mathbb{R}$ (different notation for what we stated here in (2)). Evaluate this series expression for $\theta = \pi$. The series converges to -1, but $\cos\pi(\theta)$ is semipositive definite! Therefore, the series is incorrect.

- If I am grasping the foundational ideas of the paper correctly, what is required is the following:

It seems to me that what the authors need to do is to define the series as: For all $\theta \in \mathbb{R}$,

$$\cos\pi(\theta) \equiv \begin{cases} \sum_{n=0}^{\infty} (-1)^n \frac{(\theta)^{2n}}{(2n)!} & \left[-\frac{\pi}{2}, \frac{\pi}{2}\right] \text{ mod } \pi \\ -\sum_{n=0}^{\infty} (-1)^n \frac{(\theta)^{2n}}{(2n)!} & \left[\frac{\pi}{2}, \frac{3\pi}{2}\right] \text{ mod } \pi \end{cases}$$

This series compared with the cosine series above (2), imply that $\cos\pi(\theta) = |\cos\theta|$, also that $\cos\pi(\theta)$ is semipositive definite, continuity of the function, derivative not defined at $\frac{\pi}{2} \bmod \pi$, etc.

If $j^2 = -1$ and the product is associative, it follows that $j^3 = -j$ and $j^4 = 1$. In terms of the complex exponential function, defined here by (1),

$$\cos\pi(\theta) = \begin{cases} \text{Re}\left(e^{i\theta}\right), & \left[-\frac{\pi}{2}, \frac{\pi}{2}\right] \bmod \pi \\ \text{Re}\left(-e^{i\theta}\right), & \left[\frac{\pi}{2}, \frac{3\pi}{2}\right] \bmod \pi \end{cases}$$

Or, if the authors wish to define an exponential function, let me label it e_π, with series given by

$$\text{Re}\left(e_\pi^{j\theta}\right) = \begin{cases} \text{Re}\left(e^{i\theta}\right), & \left[-\frac{\pi}{2}, \frac{\pi}{2}\right] \bmod \pi \\ \text{Re}\left(-e^{i\theta}\right), & \left[\frac{\pi}{2}, \frac{3\pi}{2}\right] \bmod \pi \end{cases}$$

then they could write

$$\cos\pi(\theta) = \text{Re}\left(e_\pi^{j\theta}\right)$$

Notice that defining the π periodic exponential function e_π with the imaginary unit i or the imaginary unit j makes no difference.

I am deliberately leaving the other half of the definition undone. The authors have to figure out how to define the series representation so that they actually reproduce $\sin\pi(\theta)$ as defined in their eq. (5)-(6) [BTW, maybe there is a nicer way to define (5)-(6)]. They will then be able to define a π periodic complex exponential function …

- Response: The authors thank the reviewer for providing detailed comments, pointing out errors, and helping us to improve the quality of the manuscript. The authors thank the reviewer for doing some of our homework. Based on the suggestions of the reviewers, we have incorporated all required changes into the revised manuscript. We have redefined the Equations (4), (5) and (6) as

$$\cos\pi(\theta) = \begin{cases} \cos(\theta), & \text{if } \theta \in \bigcup_{n=-\infty}^{\infty} [-\frac{\pi}{2} + 2\pi n, \frac{\pi}{2} + 2\pi n], \\ -\cos(\theta), & \text{if } \theta \in \bigcup_{n=-\infty}^{\infty} (\frac{\pi}{2} + 2\pi n, \frac{3\pi}{2} + 2\pi n), \end{cases} \tag{4}$$

$$\text{and } \sin\pi(\theta) = \begin{cases} \sin(\theta), & \text{if } \theta \in \bigcup_{n=-\infty}^{\infty} [-\frac{\pi}{2} + 2\pi n, \frac{\pi}{2} + 2\pi n], \\ -\sin(\theta), & \text{if } \theta \in \bigcup_{n=-\infty}^{\infty} (\frac{\pi}{2} + 2\pi n, \frac{3\pi}{2} + 2\pi n), \end{cases} \tag{5}$$

where the intervals form a complete, non-overlapping partition of the real line as

$$\mathbb{R} = \left\{ \bigcup_{n=-\infty}^{\infty} \left[-\frac{\pi}{2} + 2n\pi, \frac{\pi}{2} + 2n\pi\right] \right\} \bigcup \left\{ \bigcup_{n=-\infty}^{\infty} \left(\frac{\pi}{2} + 2n\pi, \frac{3\pi}{2} + 2n\pi\right) \right\}. \tag{6}$$

For the elevation angles, we employ the transformation for all $\theta \in \mathbb{R}$ as follows:

$$\theta \mapsto \theta \,\hat{\text{mod}}\, \pi = \begin{cases} \theta & \text{if } \theta \in [-\pi/2, \pi/2], \\ \theta - m\pi & \text{if } \theta > \frac{\pi}{2}, \\ \theta + m\pi & \text{if } \theta < -\frac{\pi}{2}, \end{cases} \tag{26}$$

where m is the smallest positive integer such that the result lies within the interval $\left[-\frac{\pi}{2}, \frac{\pi}{2}\right]$. The standard π modulo function, $\theta \mod \pi$, produces results in the interval $[0, \pi)$ that can be further adjusted to $[-\pi/2, \pi/2)$ or $(-\pi/2, \pi/2]$. Thus, $\theta \,\hat{\text{mod}}\, \pi = \theta \mod \pi$, almost everywhere. Based on the suggestions of the reviewer and above equations, we have revised Result 1 to enhance clarity and eliminate any potential contradictions or confusion. We have also added the following relations after Equation (36)

$$\cos\pi(\theta) = \begin{cases} \text{Re}\left(e^{j\theta}\right) = \sum_{n=0}^{\infty} (-1)^n \frac{(\theta)^{2n}}{(2n)!}, & \text{if } \theta \in \bigcup_{n=-\infty}^{\infty} [-\frac{\pi}{2} + 2\pi n, \frac{\pi}{2} + 2\pi n], \\ \text{Re}\left(-e^{j\theta}\right) = -\sum_{n=0}^{\infty} (-1)^n \frac{(\theta)^{2n}}{(2n)!}, & \text{if } \theta \in \bigcup_{n=-\infty}^{\infty} (\frac{\pi}{2} + 2\pi n, \frac{3\pi}{2} + 2\pi n), \end{cases}$$

$$\text{and } \sin\pi(\theta) = \begin{cases} \text{Im}\left(e^{j\theta}\right) = \sum_{n=0}^{\infty} (-1)^n \frac{(\theta)^{2n+1}}{(2n+1)!}, & \text{if } \theta \in \bigcup_{n=-\infty}^{\infty} [-\frac{\pi}{2} + 2\pi n, \frac{\pi}{2} + 2\pi n], \\ \text{Im}\left(-e^{j\theta}\right) = -\sum_{n=0}^{\infty} (-1)^n \frac{(\theta)^{2n+1}}{(2n+1)!}, & \text{if } \theta \in \bigcup_{n=-\infty}^{\infty} (\frac{\pi}{2} + 2\pi n, \frac{3\pi}{2} + 2\pi n). \end{cases}$$

Thanks and Regards,
Pushpendra Singh
(On behalf of all authors)
